# Effects of Boric Acid and Chlorhexidine as Cavity Disinfectants on Microleakage and Microshear Bond Strength in Primary Teeth

**DOI:** 10.3390/children13030417

**Published:** 2026-03-18

**Authors:** Erdem Palaz, Ayşegül Ölmez, Zeliha Hatipoğlu Palaz

**Affiliations:** 1 Private Practice, Ankara 06450, Turkey; dt.erdempalaz@gmail.com; 2 Department of Pediatric Dentistry, Faculty of Dentistry, Gazi University, Ankara 06490, Turkey; aysegul@gazi.edu.tr

**Keywords:** boric acid, chlorhexidine, dental bonding, dental leakage, disinfectants, primary teeth, shear strength

## Abstract

**Background:** Cavity disinfection is commonly performed in pediatric restorative dentistry to reduce residual bacterial contamination. Although boric acid has been proposed as a potential antimicrobial agent, its effect on marginal integrity and adhesive performance in primary teeth remains unclear. This study evaluated the effects of 3% and 5% boric acid, compared with 2% chlorhexidine (CHX), on microleakage and microshear bond strength of composite restorations in primary teeth bonded with a two-step self-etch adhesive system. **Methods:** Seventy-two extracted primary second molars were allocated to four groups (n = 18) for microleakage assessment: control, 2% CHX, 3% boric acid, and 5% boric acid. Standardized Class V cavities were prepared, disinfectants were applied for 60 s, and restorations were completed using Clearfil SE Bond and resin composite. Microleakage at occlusal and gingival margins was evaluated using dye penetration. For microshear bond strength testing, 60 primary molars (n = 15 per group) were treated similarly, and shear force was applied to bonded composite microcylinders. Data were analyzed at the *p* < 0.05 significance level. **Results:** Both boric acid groups showed significantly higher occlusal and gingival microleakage than the control and CHX groups (*p* < 0.05). Gingival microleakage was greater than occlusal microleakage in the boric acid groups (*p* < 0.05). Microshear bond strength was significantly reduced in the boric acid groups compared with the control (*p* < 0.05), whereas CHX had no significant effect. Failure modes did not differ significantly. **Conclusions:** While 2% CHX did not adversely affect adhesive performance, 3% and 5% boric acid increased microleakage and reduced bond strength. Caution is advised when using boric acid with self-etch adhesive systems in primary teeth.

## 1. Introduction

Primary teeth play a critical role in mastication, phonation, esthetics, and the guidance of permanent tooth eruption, and their preservation is essential for maintaining oral health and normal craniofacial development in children. Restorative treatment in primary teeth aims not only to remove infected carious tissue but also to prevent secondary caries and ensure long-term marginal integrity [1]. However, conventional caries removal strategies have certain limitations in clinical practice. It has been demonstrated that a solely mechanical caries excavation approach is often insufficient to achieve a completely caries-free cavity, as residual microorganisms may remain within dentinal tubules and the smear layer. These residual bacteria may contribute to postoperative sensitivity, marginal discoloration, microleakage, and secondary caries [2].

Achieving durable adhesion in primary teeth is further complicated by the structural and chemical differences between primary and permanent dentin. Bond strength has been reported to be lower in primary teeth due to their lower inorganic and higher organic content, as well as increased dentinal tubule density and diameter. The greater number and larger diameter of dentinal tubules may increase dentin permeability and fluid movement within the tubules, which can interfere with adhesive infiltration and polymerization. As a result, the formation and stability of the hybrid layer may be compromised, leading to reduced bonding effectiveness [3]. Furthermore, the tubular structure of primary dentin differs from that of permanent teeth, and the dentin surface is generally less moist. As a result, dilution of the applied acid by dentinal fluid is more limited, leading to a more rapid and deeper etching effect [4]. Nör et al. [5] also reported that primary dentin is more susceptible to acid applications and that a thicker, but less stable, hybrid layer forms following acid etching, which may negatively affect bond strength. These micromorphological characteristics make primary dentin more vulnerable to over-demineralization and compromise resin infiltration, thereby posing a challenge for reliable adhesive bonding [3].

Although cavity disinfectants cannot overcome the intrinsic structural limitations of primary dentin, their use has been advocated as an adjunct to restorative procedures to reduce the residual microbial load following caries removal. By reducing the number of remaining bacteria in the cavity, disinfectants may improve restoration longevity and reduce the risk of secondary caries, particularly in pediatric dentistry, where caries progression can be rapid [6]. An ideal cavity disinfectant should effectively eliminate residual bacteria without adversely affecting the bonding performance of adhesive systems or the integrity of the hybrid layer [7].

Chlorhexidine (CHX) is one of the most commonly used cavity disinfectants in restorative dentistry due to its broad-spectrum antimicrobial activity and substantivity. In addition to reducing residual bacterial load, CHX has been reported to inhibit dentinal matrix metalloproteinases, thereby helping preserve the hybrid layer and potentially improving bond durability [8,9]. Despite these advantages, CHX has some drawbacks; it has a limited penetration depth in deep cavities, and it may cause staining of teeth and alteration of taste in some cases [10].

Studies on primary teeth have reported conflicting results regarding the effect of CHX on adhesive performance. Studies investigating the influence of 2% CHX on adhesive performance in primary dentin have reported inconsistent findings; while some studies have suggested that CHX application may increase microleakage or reduce bond strength, others have reported no significant effect on bonding performance [11,12]. Therefore, the influence of CHX on the adhesive performance of restorative materials in primary teeth remains controversial.

Boric acid has recently attracted attention in dentistry because of its antimicrobial, antifungal, and biocompatible properties [13]. It has been proposed as an alternative cavity disinfectant and has demonstrated promising antimicrobial efficacy [14]. However, the available literature regarding its effects on adhesive bonding is limited and largely restricted to permanent teeth [13,15]. Moreover, the influence of boric acid on microleakage and bond strength in primary teeth remains unclear. Given the distinct structural characteristics of primary dentin, findings from permanent teeth cannot be directly extrapolated to pediatric populations.

To the best of our knowledge, there is a scarcity of studies simultaneously evaluating the effects of boric acid and CHX on both microleakage and microshear bond strength in primary teeth. Thus, the present in vitro study aimed to investigate the effects of 3% and 5% boric acid and 2% CHX, used as cavity disinfectants, on microleakage and microshear bond strength of composite restorations in primary teeth bonded with a two-step self-etch adhesive system. The null hypotheses were that (1) the application of boric acid or CHX would not affect microleakage and (2) would not influence microshear bond strength in primary teeth.

## 2. Materials and Methods

Ethical approval was obtained from the Clinical Research Ethics Committee of Gazi University Faculty of Dentistry, with decision dated 11 July 2019 and numbered E.23592. Primary second molars were collected from children aged 9–12 years who applied to the Department of Pediatric Dentistry at the Faculty of Dentistry, Gazi University. After visual inspection, only teeth with intact crown enamel and free of restorations, fractures, caries, or any structural defects were included in the study. Teeth presenting any of these conditions were excluded. Two pediatric dentists selected teeth by visual inspection, with at least 7 years of clinical experience. All restorative procedures, including cavity preparation and adhesive application, were performed by one of these dentists, following a standardized protocol to minimize operator-related variability.

After extraction, the teeth were cleaned of plaque and soft tissue debris under running water, disinfected in 0.5% chloramine-T trihydrate for 1 week, and stored in distilled water at −20 °C for up to 1 month [16].

### 2.1. Microleakage Test

Standard Class V cavities were prepared on the buccal surfaces of 72 primary molars using an FG-101 diamond bur (Intensive, SeDenta, İstanbul, Turkey) under water cooling (2 mm height, 3 mm width, and 2 mm depth), with the gingival margin positioned 0.5 mm coronal to the cemento-enamel junction. Cavity dimensions were verified using a millimeter-marked periodontal probe during preparation to ensure standardization. Specimens that did not meet the intended cavity dimensions due to over-preparation were excluded and replaced to maintain standardization among the samples.

The teeth were then randomly allocated into four groups:

Group C: Control (n = 18)
Group CHX: 2% Chlorhexidine (Cavity Cleanser, Bisco, Anaheim, CA, USA) (n = 18)
Group B3: 3% boric acid (n = 18)
Group B4: 5% boric acid (n = 18)

The dentin surface was treated with the assigned disinfectant for 60 s, and excess moisture was removed with absorbent paper. In the control group, distilled water was applied for 60 s. (The physicochemical characteristics of the disinfectant agents were considered in the experimental design. Boric acid is a weak Lewis acid with a pKa value of approximately 9.24 in aqueous solution. The pH of the boric acid solutions used in this study was approximately 5.1 for the 3% solution and 4.8 for the 5% solution. The pH of the 2% chlorhexidine solution was within the manufacturer’s specified range of 5.5–7.0. All experimental procedures were conducted under standard laboratory conditions at room temperature (approximately 23–25 °C). Clearfil SE Bond (Kuraray, Tokyo, Japan) was then applied according to the manufacturer’s instructions: the primer was applied for 20 s and gently air-dried for approximately 10 s to allow solvent evaporation; the air-spray tip was positioned approximately 5 mm from the tooth surface. Subsequently, the bonding agent was applied, carefully air-thinned for approximately 10 s to obtain a uniform adhesive layer, and light-cured for 10 s with an LED unit operating at 500 mW/cm^2^ (Woodpecker, Guilin, China).

Filtek Z250 Universal Composite (3M ESPE, Irvine, CA, USA) was placed incrementally, with each increment approximately 2 mm thick, and each layer was light-cured for 20 s using the same LED unit. After completion, excess material was removed with FG-5205 finishing burs (Intensive, SeDenta, Istanbul, Turkey), followed by polishing with discs and rubber points.

All restorative procedures, including cavity preparation, disinfectant application, adhesive procedures, and composite placement, were performed by a single operator with at least 7 years of clinical experience in pediatric dentistry. The procedures were carried out according to a standardized protocol to minimize operator-related variability and ensure consistency among all specimens. Since all restorations were performed by the same operator following the same protocol, the time spent per restoration was considered comparable across specimens.

After restoration, the teeth were stored in distilled water for 24 h. Two coats of nail varnish were applied, leaving the restoration and a 1 mm perimeter exposed, followed by immersion in 1% methylene blue for 4 h. The teeth were then rinsed and embedded in acrylic resin blocks. Acrylic blocks were stored in distilled water at 24 °C for 24 h and then sectioned into three longitudinal buccolingual slices per specimen using a microcut device (ATM Brillant 210, GmbH, Mammelzen, Germany). Microleakage was evaluated under a light microscope (Leica DM 4000 M, Wetzlar, Germany), which shows the images of dye penetration at the tooth–restoration interface, and scores were recorded separately for the occlusal and gingival margins as follows:

0 = No dye penetration
1 = Dye penetration up to half of the cavity wall
2 = Dye penetration beyond half of the cavity wall
3 = Dye penetration up to the cavity floor
4 = Dye penetration partially or completely reaches the pulp

### 2.2. Microshear Bond Strength Test

**Figure 1:**
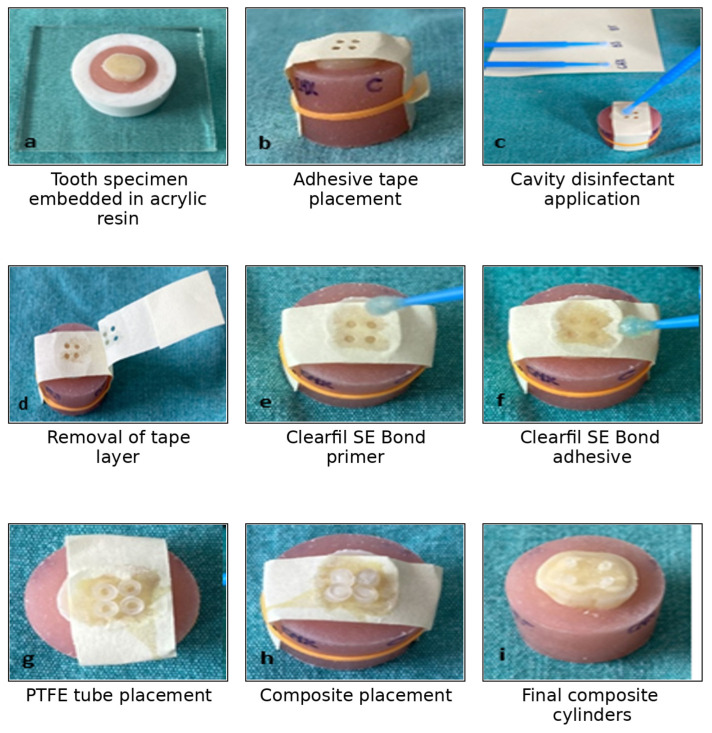
Preparation of tooth specimens.

A total of 60 extracted primary molars were used for the microshear bond strength test (n = 15 per group). The occlusal surfaces were ground under running water using a diamond disc to obtain a flat dentin surface. The teeth were then embedded in acrylic resin within polypropylene molds (14.5 mm diameter), leaving the dentin surface exposed above the acrylic level. To standardize the bonding areas, adhesive tapes with four circular holes (1 mm in diameter), prepared using a rubber dam punch, were placed over the dentin surface. These holes defined the bonding sites for the composite microcylinders (Figure 1). The specimens were then randomly divided into four groups according to the disinfectant applied:

Group C: Control (n = 15)
Group CHX: Chlorhexidine (Cavity Cleanser, Bisco, Anaheim, CA, USA) (n = 15)
Group B3: 3% boric acid (n = 15)
Group B5: 5% boric acid (n = 15)

The disinfectant solutions were applied to the dentin surface through the holes in the adhesive tape for 60 s, according to the group assignment. After disinfectant application, the adhesive tape layer was carefully removed, and the adhesive system Clearfil SE Bond (Kuraray, Tokyo, Japan) was applied according to the manufacturer’s instructions. Polytetrafluoroethylene (PTFE) tubes (1 mm inner diameter, 2 mm outer diameter, and 2 mm height) were then positioned over the predefined bonding areas and filled with Filtek Z250 Universal Composite (3M ESPE, USA). The composite was light-cured for 20 s, creating four composite microcylinders on each dentin surface.

After storage in distilled water for 24 h, teeth were mounted in a Micro-Shear Bond Tester (Bisco, Anaheim, CA, USA) with a chisel-shaped blade positioned as close as possible to the dentin–adhesive interface. A shear force was applied at a crosshead speed of 0.5 mm/min until failure. Shear bond strength values were calculated in MPa using the formula f = F/πr^2^. Failure modes were evaluated under a stereomicroscope at 40× magnification.

Statistical analysis was performed using IBM SPSS Statistics v22 (IBM Corporation, Armonk, NY, USA). Normality was assessed using the Kolmogorov–Smirnov and Shapiro–Wilk tests. Parametric data were analyzed using one-way ANOVA with Tukey’s post hoc test. In contrast, non-parametric data were analyzed using the Kruskal–Wallis test with Dunn’s post hoc test and the Wilcoxon signed-rank test. Categorical data were analyzed using the chi-square test. Statistical significance was set at *p* < 0.05.

## 3. Results

The occlusal microleakage levels in both Group B3 and Group B5 were significantly higher than those in Group C and Group CHX (*p* < 0.05). No statistically significant difference was observed between Group C and Group CHX (*p* > 0.05), nor between Group B3 and Group B5 (*p* > 0.05). The occlusal microleakage results are presented in Table 1.

The gingival microleakage levels in both Group B3 and Group B5 were significantly higher than those in Group C and Group CHX (*p* < 0.05). No statistically significant difference was observed between Group C and Group CHX (*p* > 0.05), nor between Group B3 and Group B5 (*p* > 0.05). The gingival microleakage results are presented in Table 2.

**Figure 2:**
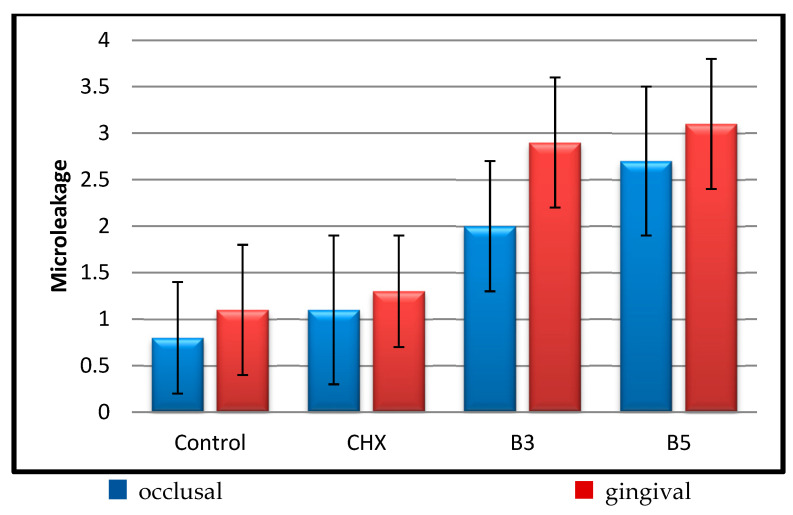
Evaluation of occlusal and gingival microleakage scores.

No statistically significant difference was observed between the occlusal and gingival microleakage levels in Group Control (*p* = 0.166) and Group CHX (*p* = 0.248) (*p* > 0.05). In contrast, the gingival microleakage level was significantly higher than the occlusal level in both Group B3 (*p* = 0.001) and Group B5 (*p* = 0.020) (*p* < 0.05). The corresponding microleakage scores are shown in Figure 2.

**Figure 3:**
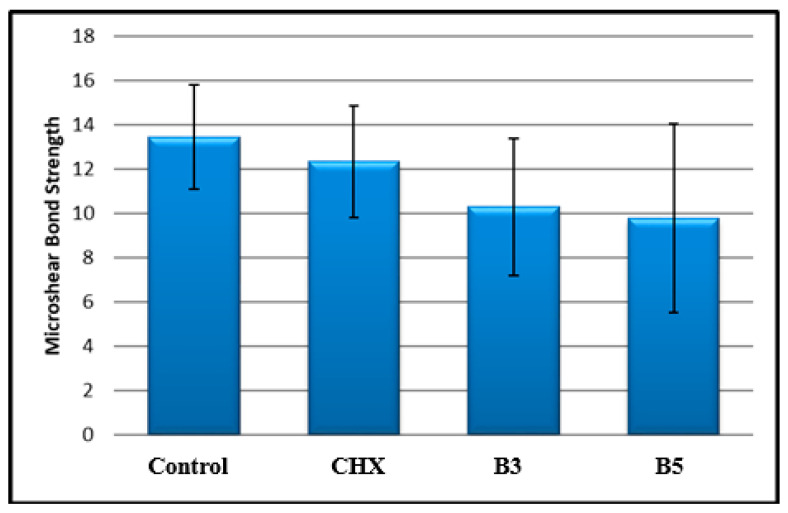
Distribution of microshear bond strength scores among the groups.

There was a statistically significant difference in mean shear bond strength among the groups (*p* < 0.05). No significant difference was found between Group C and Group CHX; however, the mean shear bond strength of Group C was significantly higher than that of Group B3 (*p* = 0.038) and Group B5 (*p* = 0.012) (*p* < 0.05). No other significant differences were observed among the groups (*p* > 0.05). The shear bond strength values are shown in Figure 3.

**Figure 4:**
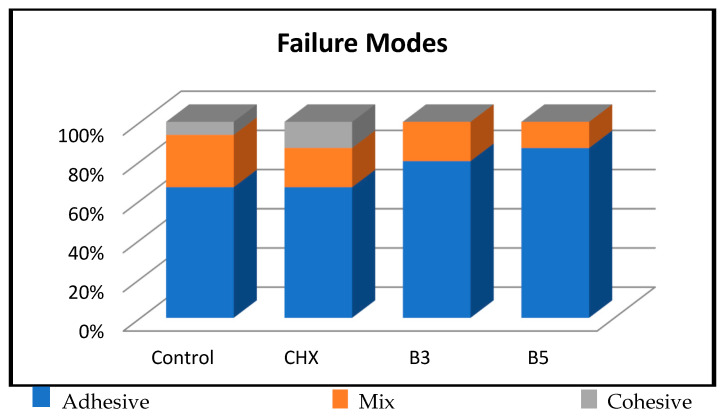
The distribution of failure modes.

There was no statistically significant difference among the groups in terms of the distribution of failure modes (*p* > 0.05). Adhesive failure was observed in 66.7% of Group C, 66.7% of Group CHX, 80% of Group B3, and 86.7% of Group B5. Mixed failure was observed in 26.7% of Group C, 20% of Group CHX, 20% of Group B3, and 13.3% of Group B5. Cohesive failure was observed in 6.7% of Group C and 13.3% of Group CHX; however, no cohesive failure was detected in the boric acid groups. The distribution of failure modes among the groups is shown in Figure 4.

## 4. Discussion

The use of cavity disinfectants has gained increasing attention in pediatric restorative dentistry due to their ability to reduce residual bacteria and potentially enhance the longevity of restorations [6]. However, their clinical effectiveness depends not only on antimicrobial activity but also on their interactions with primary dentin, adhesive systems, and restorative materials, which may influence bonding performance by altering dentin surface characteristics or the stability of the hybrid layer [7,17]. In the context of chemical cavity disinfection, CHX is a biologically active biguanide that exerts a strong antimicrobial effect by interacting with amino acids in the dentin matrix and supports the long-term stability of the resin–dentin interface by inhibiting matrix metalloproteinases (MMPs) [18,19]. While CHX has been extensively recognized for its antimicrobial and MMP-inhibitory properties, boric acid has recently emerged as a potential alternative due to its antimicrobial effectiveness and favorable biocompatibility profile [13,20].

Although the number of studies evaluating the effect of cavity disinfectant application on microleakage in primary teeth is limited, available evidence suggests that such applications may influence marginal sealing. Tulunoğlu et al. [21], in an in vivo study on exfoliating primary canines, reported that the application of 2% CHX as a cavity disinfectant increased the marginal microleakage of Class V composite restorations when used with both a two-step etch-and-rinse adhesive system (Prime & Bond, Dentsply, Charlotte, NC, USA) and a two-step self-etch adhesive system (Syntac, Ivoclar, Schaan, Liechtenstein). Similarly, Memarpour et al. [11] found that in Class V restorations of primary canines, the application of 2% CHX after acid etching and before bonding resulted in increased microleakage when used with either a self-etch adhesive (Clearfil Protect Bond, Kuraray Noritake Dental Inc., Tokyo, Japan) or an etch-and-rinse adhesive system (Adper Single Bond 2, 3M ESPE). Also, Haralur et al. [22] and Kimyai et al. [23] reported that CHX may adversely affect dentine by reducing calcium content and hardness and by compromising the bonding and sealing ability of adhesive restorations, potentially increasing microleakage. In addition, a recent study comparing cavity disinfection methods in primary teeth found that CHX application was associated with increased microleakage compared with laser-based disinfection [24].

In the present study, however, no significant difference in microleakage was observed between the control and CHX groups. When occlusal and gingival microleakage scores were evaluated within groups, neither the control nor the CHX group demonstrated a significant increase in gingival microleakage compared with occlusal margins. This discrepancy with previously reported findings may be attributed to differences in adhesive protocols, restorative materials, and experimental conditions. In line with this interpretation, it has been suggested that the effect of cavity disinfectants on marginal sealing is material-dependent and closely related to their interactions with specific dentin bonding systems and their ability to effectively seal dentin, rather than the disinfectant alone [21]. Similarly, Salama et al. [25] reported that, in line with the present study’s findings, the use of CHX as a cavity disinfectant in primary teeth did not significantly affect restorative microleakage.

Evidence regarding the effects of boric acid on adhesive bonding and marginal integrity in primary teeth remains limited. Previous studies conducted on permanent teeth have suggested that boric acid, particularly at concentrations of 3% and 5%, may be a suitable cavity disinfectant. Cangül et al. [26] reported that these concentrations were associated with the lowest microleakage values in permanent canine teeth. In addition, Aktürk et al. [13] and Culhaoğlu et al. [20] reported that 3% and 5% boric acid solutions could be viable alternatives to CHX as cavity disinfectants. Therefore, 3% and 5% boric acid concentrations were selected for use in the present study. However, contrary to these findings, the present study demonstrated that the application of 3% and 5% boric acid as a cavity disinfectant increased microleakage at both the occlusal and gingival margins. In addition, the distinct structural characteristics of primary dentin may influence the interaction between disinfectant agents and adhesive systems. Consequently, the response of primary dentin to boric acid treatment may differ from that observed in permanent teeth.

In the present study, a self-etch adhesive system was used, eliminating the separate etching, rinsing, and drying steps and thereby reducing operator-dependent variability [27,28]. Owing to their milder interaction with dentin and their ability to effectively seal dentinal tubules, self-etch adhesive systems also contribute to a reduced incidence of postoperative sensitivity. Considering these clinical advantages—particularly relevant in pediatric dentistry, where moisture control can be challenging—a simplified, less technique-sensitive two-step self-etch adhesive system, Clearfil SE Bond (Kuraray, Tokyo, Japan), was selected for this study.

The use of 2% CHX in combination with a two-step self-etch adhesive system in the present study was based on both methodological and clinical considerations. Although chlorhexidine has frequently been reported to produce favorable outcomes when used with etch-and-rinse adhesive systems, its use with self-etch adhesive systems may also be considered appropriate depending on the adhesive strategy and the structural characteristics of dentin [12]. In addition, self-etch systems were preferred in the present study because they simplify the bonding procedure by eliminating separate etching and rinsing steps, thereby reducing technique sensitivity and operator-related variability. This approach is particularly relevant in pediatric dentistry, where moisture control and patient cooperation may be limited. Another possible explanation for the stable bonding performance observed in the CHX group may be the chemical interactions provided by functional monomers in self-etch adhesive systems. The 2% CHX concentration was selected because it is the most commonly used concentration in both dental research and clinical practice as a cavity disinfectant, and its antimicrobial efficacy and substantivity have been well documented. Additionally, the 2% concentration is considered biocompatible and has an acceptable toxicological profile [29]. At low concentrations, CHX exerts a bacteriostatic effect by altering the osmotic balance of bacterial cells. In contrast, at higher concentrations, it shows bactericidal activity by increasing membrane permeability and precipitating cytoplasmic components [30].

Nevertheless, the structural and compositional characteristics of primary tooth dentin may help explain the unfavorable outcomes observed following boric acid application. Primary dentin is more susceptible to acidic agents due to its higher organic content, larger and more numerous dentinal tubules, and lower inherent moisture, making it more prone to excessive demineralization after boric acid treatment [3]. This condition may disrupt the balance required for optimal adhesive penetration, leading to inadequate resin infiltration. Particularly when two-step self-etch adhesive systems are used, an over-demineralized dentin surface may result in a thicker, but less stable, hybrid layer. As reported by Nör et al. [5], such hybrid layers are associated with reduced bond strength. This mechanism may represent one possible explanation for the increased microleakage and decreased bond strength observed in the groups treated with 3% and 5% boric acid in the present study. Previous studies have demonstrated that applying 2% CHX to primary dentin, when used in conjunction with self-etch adhesive systems, does not adversely affect immediate bond strength [12,31,32]. In the present study, the use of 2% CHX as a cavity disinfectant in combination with a self-etch adhesive system likewise did not result in a significant change in microshear bond strength. Similarly, Ersin et al. [33] and Ricci et al. [34] reported that the application of 2% CHX in primary teeth did not affect immediate bond strength. Öznurhan et al. [35] also found that applying 2% CHX prior to etch-and-rinse adhesives in primary dentin did not significantly influence bond strength. However, the application of 2% CHX has also been associated with reduced bond strength under certain conditions, depending on the restorative material and adhesive protocol used [36]. Variations in bond strength values may be attributed to differences in the CHX application protocol (e.g., whether it is applied before or after the etching or rinsing step), the hydrophilic characteristics of dentin, the method of material application, and the duration of exposure [19].

Several studies have evaluated the effects of boric acid as a cavity disinfectant on bond strength in permanent teeth [14,15]. Ercan et al. [15] examined the influence of ozone, a 2% CHX solution, and a 5% boric acid solution on the shear bond strength of one-step self-etch adhesive systems in permanent molars. They reported that the shear bond strength of the control group was comparable to that of the ozone group, whereas the CHX and boric acid groups showed comparable bond strength values, but significantly lower than those of the control group. Conversely, Aktürk et al. [13] investigated the effects of three different cavity disinfectants and three adhesive systems on microshear bond strength. In a study conducted on permanent third molars using self-etch and universal adhesives, the 2% CHX solution demonstrated the highest bond strength compared with ozonated water, boric acid, and the control group. According to the present study, the use of 3% and 5% boric acid solutions in primary teeth, in combination with self-etch adhesive systems, resulted in reduced bond strength. The main difference between the present findings and those of previous studies is that earlier investigations were conducted on permanent teeth. The results of this study suggest that, due to boric acid’s organic, acidic nature, its application may lead to thickening of the hybrid layer and alterations in the smear layer’s structure. These changes may adversely affect adequate and homogeneous resin infiltration into dentin, thereby contributing to decreased microshear bond strength values.

In the present study, no statistically significant differences were observed among the groups regarding failure mode distribution. Nevertheless, adhesive failures were more prevalent in the 3% and 5% boric acid groups, while dentin cohesive failures occurred more frequently in the control and 2% CHX groups. This pattern suggests that bond strength was higher in the control and CHX groups compared with the boric acid groups. Consistent with these findings, the first null hypothesis was partially rejected: the application of boric acid significantly increased both occlusal and gingival microleakage, whereas CHX did not. Similarly, the second null hypothesis was partially rejected, as boric acid significantly reduced microshear bond strength in primary teeth, whereas CHX showed no significant effect on bond strength.

In interpreting these findings, it should also be noted that the antimicrobial effects attributed to cavity disinfectants are primarily due to the agents’ chemical properties. The LED curing unit used in this study emits visible blue light and is mainly intended to initiate the polymerization of the restorative material rather than to provide antimicrobial activity. In addition, the same light-curing protocol was applied to all groups; therefore, any potential influence of the curing light would have affected all specimens equally and would not have influenced the comparison among the experimental groups.

This study has several limitations that should be considered when interpreting the findings. Although dye penetration is widely used for microleakage assessment, it provides only a two-dimensional evaluation and may not accurately reflect the true extent of leakage. More advanced techniques—such as light microscopy, scanning and transmission electron microscopy, and three-dimensional methods including micro-computed tomography (µCT), confocal laser scanning microscopy, and optical coherence tomography—have been shown to offer superior visualization of microleakage [37]. Additionally, in vitro studies cannot fully replicate the oral environment and may therefore fail to encompass all relevant clinical variables.

Another limitation is that the study was conducted under controlled laboratory conditions using a single adhesive system and a limited number of disinfectant concentrations, which may restrict the generalizability of the findings. Given the limited number of studies investigating the use of boric acid as a cavity disinfectant in primary teeth, further research is warranted. Future studies should incorporate larger sample sizes, diverse adhesive strategies, and long-term aging protocols to simulate clinical conditions better and clarify the long-term effects of boric acid on adhesive performance.

## 5. Conclusions

Based on the findings of the present study, the application of CHX as a cavity disinfectant before the use of self-etch adhesive systems did not reduce bond strength in primary teeth. Furthermore, no statistically significant increase in microleakage was observed following CHX application. Its ability to inhibit secondary caries and reduce postoperative sensitivity may therefore contribute to the longevity of restorations. In contrast, the use of boric acid solutions in primary teeth, combined with self-etch adhesive systems, was associated with increased microleakage and reduced bond strength. Nevertheless, further in vitro and clinical studies are required to confirm these findings.

## Figures and Tables

**Table 1:** Evaluation of occlusal microleakage.

	C	CHX	B3	B5	
Occlusal Microleakage	n (%)	n (%)	n (%)	n (%)	*p*
0	6 (%33.3)	5 (%27.8)	0 (%0)	0 (%0)	
1	10 (%55.6)	7 (%38.9)	4 (%22.2)	1 (%5.6)	
2	2 (%11.1)	6 (%33.3)	10 (%55.6)	6 (%33.3)	0.000 *
3	0 (%0)	0 (%0)	4 (%22.2)	8 (%44.4)	
4	0 (%0)	0 (%0)	0 (%0)	3 (%16.7)	

Chi-square test. * *p* < 0.05. 0 = No dye penetration. 1 = Dye penetration up to half of the cavity wall. 2 = Dye penetration beyond half of the cavity wall. 3 = Dye penetration up to the cavity floor. 4 = Dye penetration partially or completely reaches the pulp.

**Table 2:** Evaluation of gingival microleakage.

	Control	CHX	B3	B5	
Gingival Microleakage	n (%)	n (%)	n (%)	n (%)	*p*
0	4 (%22.2)	1 (%5.6)	0 (%0)	0 (%0)	
1	9 (%50)	11 (%61.1)	0 (%0)	0 (%0)	
2	5 (%27.8)	6 (%33.3)	5 (%27.8)	3 (%16.7)	0.000 *
3	0 (%0)	0 (%0)	10 (%55.6)	10 (%55.6)	
4	0 (%0)	0 (%0)	3 (%16.7)	5 (%27.8)	

Chi-square test. * *p* < 0.05. 0 = No dye penetration. 1 = Dye penetration up to half of the cavity wall. 2 = Dye penetration beyond half of the cavity wall. 3 = Dye penetration up to the cavity floor. 4 = Dye penetration partially or completely reaching the pulp.

## Data Availability

The original contributions presented in the study are included in the article. Further inquiries can be directed to the corresponding author.
